# IceSense Proof of Concept: Calibrating an Instrumented Figure Skating Blade to Measure On-Ice Forces

**DOI:** 10.3390/s20247082

**Published:** 2020-12-10

**Authors:** Sarah Ridge, Dustin Bruening, Steven Charles, Cody Stahl, Daniel Smith, Riley Reynolds, Brandon Adamo, Blake Harper, Chris Adair, Preston Manwaring, Deborah King

**Affiliations:** 1 Department of Exercise Sciences, Brigham Young University, Provo, UT 84602, USA; dabruening@byu.edu; 2 Department of Mechanical Engineering, Brigham Young University, Provo, UT 84602, USA; skcharles@byu.edu (S.C.); dannysmith@gatech.edu (D.S.); rileyreynolds1218@gmail.com (R.R.); jbadamo14@gmail.com (B.A.); blake.harper@byu.edu (B.H.); chriswadair@byu.edu (C.A.); 3 Department of Exercise Science and Athletic Training, Ithaca College, Ithaca, NY 14850, USA; cstahl@uccs.edu (C.S.); dking@ithaca.edu (D.K.); 4 Blue Elm Consulting, Farmington, UT 84025, USA; preston.k.manwaring@dartmouth.edu

**Keywords:** force, validation, calibration, figure skating, on ice

## Abstract

Competitive figure skaters often suffer from overuse injuries, which may be due to the high impact forces endured during jump repetitions performed in practice and competition. However, to date, forces during on-ice figure skating have not been quantified due to technological limitations. The purpose of this study was to determine the optimal calibration procedure for a previously developed instrumented figure skating blade (IceSense). Initial calibration was performed by collecting data from the blade while 11 skaters performed off-ice jumps, landing on a force plate in the lab. However, mean peak force measurements from the blade were greater than the desired error threshold of ±10%. Therefore, we designed a series of controlled experiments which included measuring forces from a load cell rigidly attached to the top of the blade concurrently with strain data from the strain gauges on the blade. Forces were applied to the blade by adding weight to a drop tower or by manually applying force in a quasi-static manner. Both methods showed similar accuracy, though using the drop tower allowed precise standardization. Therefore, calibration was performed using the weighted drop method. This calibration was applied to strain gauge data from out-of-sample drop trials, resulting in acceptable estimates of peak force (less than 10% error). Using this calibration, we collected data on one figure skater and present results from an exemplar on-ice double flip jump. Using the IceSense device to quantify on-ice forces in a research setting may help inform training, technique, and equipment design.

## 1. Introduction

Many competitive figure skaters suffer from overuse injuries, such as tendinitis, stress fractures, patellofemoral syndrome, muscle strains, and low-back pain [1,2,3,4]. It has been suggested that many of these injuries are related to both the high number of jumps skaters perform in practice and to the rigidity of figure skating boots [5]. Elite figure skaters train extensively, performing over 50 multi-revolution jumps daily [6]. As difficult jumps have become more prevalent throughout the past century of figure skating, the skating boot has also become stiffer to provide landing stability [5]. However, it is thought that this increased stiffness could contribute to injuries by limiting joint motion, thus reducing the ability of the body to absorb high impact forces [5,7,8]. The frequency and magnitude of impact forces during landings have long been associated with overuse injuries in other sports [5,8,9]. It is imperative to be able to quantify on-ice forces so that future research can determine whether any relationships exist between figure skating landing mechanics, boot stiffness, and injury incidence.

While many skaters, coaches, and trainers intuitively recognize that skaters experience large forces during jumping and landing, addressing the relationship between these impact forces and overuse injuries is difficult without accurate force measurement methods. Since typical force measurement tools cannot be used on the ice, a few studies have attempted to estimate these forces indirectly. Bruening and Richards [6] and Kho [10] both used off-ice jump simulations (landing on a force plate in a lab), finding that skaters may experience peak vertical forces of approximately 5 times their own body weight during each jump landing. Other researchers have attempted to capture on-ice landing impacts using either pressure sensing insoles [11] or tibial-mounted accelerometers [12]. However, traditional pressure sensing insoles are difficult to incorporate in a skating boot due to the extremely tight fit of the boots and the bulkiness of the insole and related electronics, which could interfere with the performance of highly technical skating skills. They also only measure vertical force and have limited sampling rates. High sampling rates may be critical since the transition from toe to heel contact lasts approximately 50 ms and key features such as peak force and loading rate comprise small portions of that time frame. Commercial accelerometers do have high sampling rates but do not capture the impact forces, only the associated accelerations. While advances in these products may make them suitable for this application in the near future, alternate and complementary measurements that capture multi axis forces at high sample rates will still be critical.

Accurate on-ice measurements of force have not yet been reported. Previously, we reported on the design of a device that is lightweight, small in size, and water resistant [13]. This force measurement system consists of strain gauges on the three stanchions that support the skate blade, along with Wheatstone bridge circuit boards and a data acquisition device mounted under the boot. In addition to the design, we presented preliminary off-ice data from a single skater landing on a force. The relationship between the voltage output of the strain gauges and the force on the blade was calibrated using a static calibration (fixed weights) and a limited dynamic calibration (using force data from one landing trial to calculate forces from one different landing trial). This calibration showed an RMS error of 128 N over a 0.55 s impact period. While this appeared promising, further testing is needed to determine how generalizable the calibration is and how well it captures the peak force, which may be directly related to injury. Although the goal of this project involves only building enough devices to apply to a range of blade sizes for research purposes, it is clear that each device that is created will have to be calibrated separately. It would be ideal, however, if the system can work on multiple participants without having to perform different calibrations for each participant. Therefore, the purpose of the current study was to further develop calibration procedures to create a robust, generalizable calibration method in order to accurately measure force during on-ice landings.

## 2. Methods/Results

### 2.1. Overview

**Figure 1:**
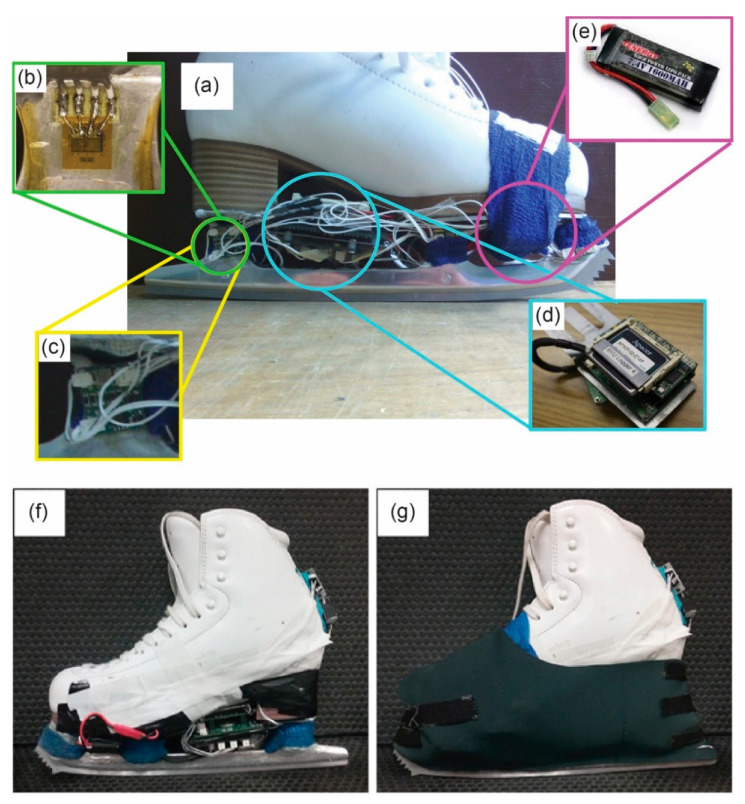
Instrumented figure skate. (**a**) Exposed electronics, consisting of strain gauges (**b**) mounted to each side of each stanchion, Wheatstone bridge boards (**c**) to measure changes in the strain gauges, signal-conditioning and data logger circuits (**d**), and the lithium polymer battery (**e**) powering the system. To protect the system against vibration and ice/water, the electronics were fastened to the boot using brackets and tape (**f**) and covered with a neoprene cover (**g**).

The system used in the present study (Figure 1) is an improved version of the IceSense system described previously [13] (see Supplementary Material for a description of the improvements).

Based on the promising preliminary test results presented in our previous paper [13], the first step for calibrating and validating the system was to use off-ice jumps onto a force plate (called “force plate validation”). The goals of this calibration validation were to determine the number and type of landings to include in calibration for accurate peak force estimation. We also wanted to estimate the accuracy of out-of-sample testing using cross-validation. These results showed unacceptable error magnitudes (greater than 10% of the skater’s body weight, or 50 N based on a small skater) in out-of-sample peak force estimation. We then tested a more controlled loading environment (called “load cell validation”) to generate the calibration data. In this testing, we sought to determine the effect of loading type, blade angle, and horizontal strain on calibration accuracy. We present first the force plate validation methods and results, followed by the load cell validation methods and results, and conclude with an example of on-ice testing.

### 2.2. Force Plate Validation

Eleven female skaters with no self-reported lower-extremity injuries or contraindications for jumping gave their written informed consent (as approved by the Ithaca College Humans Subjects Review Board, study #HSR 0215-04) to participate in the force plate validation. Skaters had to wear a 25.5 cm boot (Risport R3, Risport, Treviso, Italy) and land on their right foot. Skating level varied from juvenile to senior. Each skater wore the same pair of skating boots with a 24.8 cm blade (Jackson Ultima, Waterloo, ON, Canada). The blade of the right boot was instrumented as described previously [13], with the improvements listed. Blade strain data were collected at 1000 Hz.

**Figure 2:**
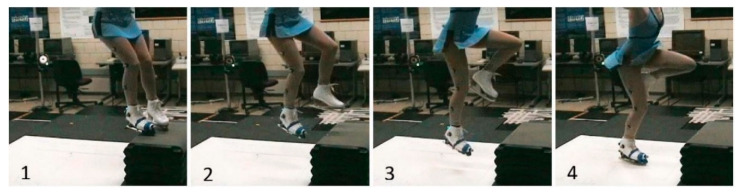
Force plate validation setup. Skaters performed drop landings from a box onto two force plates covered with HDPE artificial ice. Time progresses from **left** (1) to **right** (4).

Sixteen retro-reflective markers were placed on anatomical landmarks of the skater (Figure 2) using the Plug-in-Gait marker set (Vicon, Centennial, CO, USA), with toe and heel marker placements transferred to corresponding boot landmarks. A 7-camera motion capture system (MX3+, Vicon, Centennial, CO, USA) recording at 250 fps was synchronized with 2 force plates (Optima OR6-6-OP, AMTI, Watertown, MA, USA) recording at 1000 Hz to capture the skaters’ motions. Skaters performed landings (Figure 2) onto two adjacent calibrated force plates covered with a single piece of 1.2 cm thick HDPE plastic to create an artificial ice surface. The hardness of HDPE is of the same order of magnitude as that of ice. Specifically, the Shore D hardness of HDPE is 50–70; the Shore A hardness of ice is approximately 90 [14], which corresponds to a Shore D hardness of approximately 50 [15].

#### 2.2.1. Protocol

Each skater performed 18 to 24 landings onto the force plates for a total of 128 landings. Landings were a combination of backward hops off of a box and simulated waltz jumps. Each skater performed four backward hop landings from a height of 18 cm and four from a height of 27 cm. Twelve to 16 additional landings were completed, depending on the skater’s ability. These included waltz jumps and additional backward hops from heights of 27 cm or 36 cm. Waltz jumps required a forward take off from the left foot, driving the right foot forward with a 180° turn in the air prior to landing. Waltz jumps were performed from the box (from heights of 27 and 36 cm) or from the HDPE plastic (jumping and landing on the same surface). Each skater self-selected the maximum height from which they could safely land and whether they could complete waltz jumps on the artificial ice surface. Box heights options were chosen to replicate on-ice jump heights [6]. Skaters were allowed to practice all backward hops and waltz jumps. They rested at least 30 s between trials and several minutes between heights.

#### 2.2.2. Data Analysis

**Figure 3:**
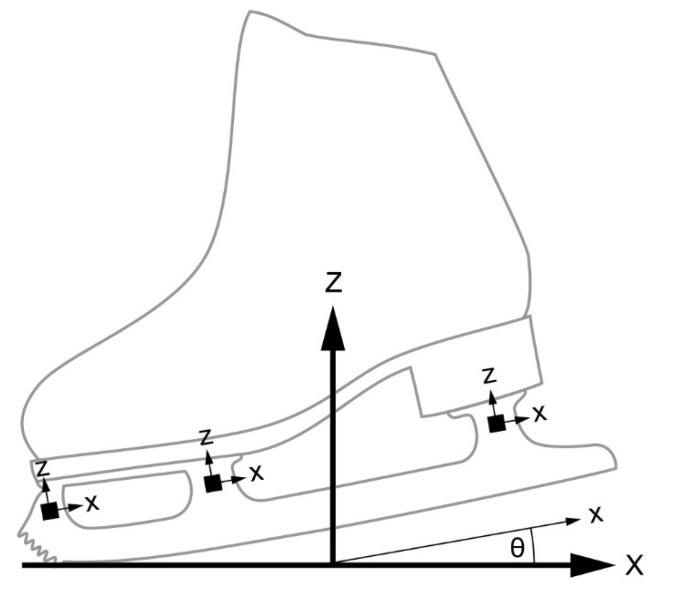
Definition of blade coordinate system (xyz) and intermediate coordinate system (XYZ). The strain gauges (represented as small black squares) were aligned with the blade coordinate system. The intermediate coordinate system is the same as the force plate frame, but rotated about the Z axis so the X axis is in the forward–backward direction of the blade.

Pre-processing: As described previously [13], horizontal and vertical strain gauges were mounted to both sides of each of the three stanchions. Each strain gauge was mounted so its x- and z axes aligned with those of the other gauges, creating a coordinate system for the blade (Figure 3). Due to practical limitations in mounting strain gauges to the thin, anterior–posterior face of the stanchions, as well as limited data acquisition channels, we did not measure strain along the y axis (medial–lateral direction). The outputs from the gauges on a stanchion were wired into Wheatstone bridges to compensate for bending, resulting in a single voltage along the *x* axis and a single voltage along the *z* axis of the blade coordinate system for each of the three stanchions: $v_{1x}$, $v_{1z}$, $v_{2x}$, $v_{2z}$, $v_{3x}$, and $v_{3z}$ (subscripts 1, 2, and 3 refer to the front, middle, and back stanchions, respectively). To calibrate the relationship between these six voltages and the force measured by the force plate (defined by coordinate system X_FP_Y_FP_Z_FP_, with Z_FP_ pointing perpendicular to the face of the force plate), we transformed these voltages into an intermediate frame (XYZ in Figure 3) whose *Z* axis was the same as the Z_FP_ axis of the force plate and whose *X* axis was in the X_FP_-Y_FP_ plane of the force plate, oriented along the forward–backward axis of the blade (i.e., the projection of the *x* axis of the blade onto the force plate). To transform the voltages into the XYZ frame, we first determined the orientation of the blade relative to the force plate coordinate system at each point in time from the motion capture data. Second, the time-varying angle between the *x* axis of the blade and the XY plane, θ (Figure 3), was low-pass filtered with a fourth-order Butterworth filter with a cut-off frequency of 50 Hz (sufficient to capture the relatively low frequency movement of human joints) and resampled at the sampling frequency of the voltages (1000 samples/s). Third, we identified the initial contact (IC) with the ice as the first increase in any of the strain gauge voltages above the flat baseline that occurred while the skater was in the air. Fourth, we zeroed each voltage by subtracting the mean voltage collected during the 100 ms prior to IC from each voltage collected during landing. Finally, the six voltages were transformed from the xz frame of the blade into the XZ plane using a rotation matrix:

$$\left[\begin{array}{c} v_{iX}\\ v_{iZ} \end{array}\right]\;=\;\;\left[\begin{array}{cc} \cos\theta & -\sin\theta \\ \sin\theta & \cos\theta \end{array}\right]\left[\begin{array}{c} v_{ix}\\ v_{iz} \end{array}\right]$$

where i = 1, 2, or 3.

Force plate data pre-processing consisted of four steps. First, we summed the vertical forces ($F_{Z}$) and the horizontal forces ($F_{X}$) from each plate. Second, we calculated the force in the X direction of the intermediate XYZ frame from the horizontal force plate forces (Figure 3). Third, we low-pass filtered the summed data with a fourth-order low-pass filter with a cut-off frequency at 20 Hz. This low cut-off frequency was determined experimentally as the frequency that removed noise but preserved the magnitudes of the peak forces. Fourth, IC was identified from the force plate data as the first instance after flight that the vertical force increased by 1 N.

Training the calibration matrix: Before determining the relationship between blade data and force plate data, all data were cropped from IC to the first local force minimum following peak force (in the low-pass filtered force plate data). The relationship between transformed voltage and force plate data was assumed to be linear:

(1) $$F_{X}\;=\;C_{X1}v_{1X}+C_{X2}v_{1Z}+C_{X3}v_{2X}+C_{X4}v_{2Z}+C_{X5}v_{3X}+C_{X6}v_{3Z}\;F_{Z}\;=\;C_{Z1}v_{1X}+C_{Z2}v_{1Z}+C_{Z3}v_{2X}+C_{Z4}v_{2Z}+C_{Z5}v_{3X}+C_{Z6}v_{3Z}$$

Since this relationship holds for data taken at different points in time, it can be written for all data points as

(2) $$\begin{array}{l} \left[\begin{array}{cc} \begin{array}{c} F_{X}\left(t_{1}\right)\\ F_{X}\left(t_{2}\right)\\ \begin{array}{c} \vdots \\ F_{X}\left(t_{n}\right) \end{array} \end{array} & \begin{array}{c} F_{Z}\left(t_{1}\right)\\ F_{Z}\left(t_{2}\right)\\ \begin{array}{c} \vdots \\ F_{Z}\left(t_{n}\right) \end{array} \end{array} \end{array}\right]\;\\ =\;[\begin{array}{cccccc} v_{1X}\left(t_{1}\right) & v_{1Z}\left(t_{1}\right) & v_{2X}\left(t_{1}\right) & v_{2Z}\left(t_{1}\right) & v_{3X}\left(t_{1}\right) & v_{3Z}\left(t_{1}\right)\\ v_{1X}\left(t_{2}\right) & v_{1Z}\left(t_{2}\right) & v_{2X}\left(t_{2}\right) & v_{2Z}\left(t_{2}\right) & v_{3X}\left(t_{2}\right) & v_{3Z}\left(t_{2}\right)\\ \vdots & \vdots & \vdots & \vdots & \vdots & \vdots \\ v_{1X}\left(t_{n}\right) & v_{1Z}\left(t_{n}\right) & v_{2X}\left(t_{n}\right) & v_{2Z}\left(t_{n}\right) & v_{3X}\left(t_{n}\right) & v_{3Z}\left(t_{n}\right) \end{array}][\begin{array}{cc} C_{X1} & C_{Z1}\\ C_{X2} & C_{Z2}\\ C_{X3} & C_{Z3}\\ C_{X4} & C_{Z4}\\ C_{X5} & C_{Z5}\\ C_{X6} & C_{Z6} \end{array}] \end{array}$$

or $F_{C}\;=\;V_{C}\;C$, where the subscript C indicates that $F_{C}$ and $V_{C}$ are the transformed force plate and blade voltage data measured *during* calibration. The coefficient matrix C was calculated by least-squares regression: $C\;=\;V_{C}{}^{†}F_{C}$, where $V_{C}{}^{†}\;=\;{\left(V_{C}{}^{T}V_{C}\right)}^{-1}V_{C}{}^{T}$ and superscript T denotes the transpose operation. Finally, with an estimate of the coefficient matrix C, the force experienced by the blade was calculated for any measured voltage matrix V as $\hat{F}\;=\;VC$. Note that $\hat{F}\;=\;\left[{\hat{F}}_{X},\;{\hat{F}}_{Z}\right]$ is in the XYZ frame.

Testing the calibration matrix: The transformed strain gauge data were multiplied by the calibration matrix, yielding the estimate of the vertical force from the strain gauge data (${\hat{F}}_{Z}$). To compare ${\hat{F}}_{Z}$ to $F_{Z}$ (the vertical force measured by the force plate), we calculated Pearson product moment correlations using 500 time points starting at IC. Peak force was also extracted. Analyses were performed using LabView (National Instruments, Austin, TX, USA).

Number of landings: First, to determine the effect of the number of landings entered into the regression on the accuracy of the calibration *within* a given participant, we created ten calibration matrices using 1, 2, …, 10 landings (including combinations of drop landings and waltz jumps) for each skater. The calibration matrices were then used to estimate vertical force from the strain gauge data for 12 of the remaining jumps of the same skater. Second, to determine the effect of the number of landings entered into the regression on the accuracy of the calibration *between* participants, we calculated three calibration matrices using 1, 5, and 10 landings from one participant. From these three calibration matrices, we estimated the vertical force during 118 landings from the remaining 10 skaters.

#### 2.2.3. Results of Force Plate Validation

Number of landings: Within-participant correlations between the vertical forces of the strain gauge and force plate data were moderate to very strong ranging from r = 0.76 to r = 0.97 with 58 to 94 percent of variation in force plate data accounted for by the blade strain gauge system. Differences in peak forces were greater than the ±50 N threshold. Mean peak differences ranged from underestimating the force plate peak force by 255 N (calibrated with 7 landings) to overestimating it by 534 N (calibrated with 1 landing). Accuracy improved with 6 or more landings, but did not meet the ±50 N standard.

When we used the calibration matrix from one participant to predict the forces of another participant, correlations were fair to very strong. Correlation coefficients between the blade and force plate vertical force curves ranged from r = 0.46 to r = 0.99. Differences in peak force were 10 to 25 times greater than the 50 N threshold. When 1, 5, and 10 landings were included in the calibration, mean peak force errors ranged from −1276 to +1011, −1129 to +567, and −1265 to +507 N, respectively, depending on the participant.

#### 2.2.4. Conclusions from Force Plate Validation

**Figure 4:**
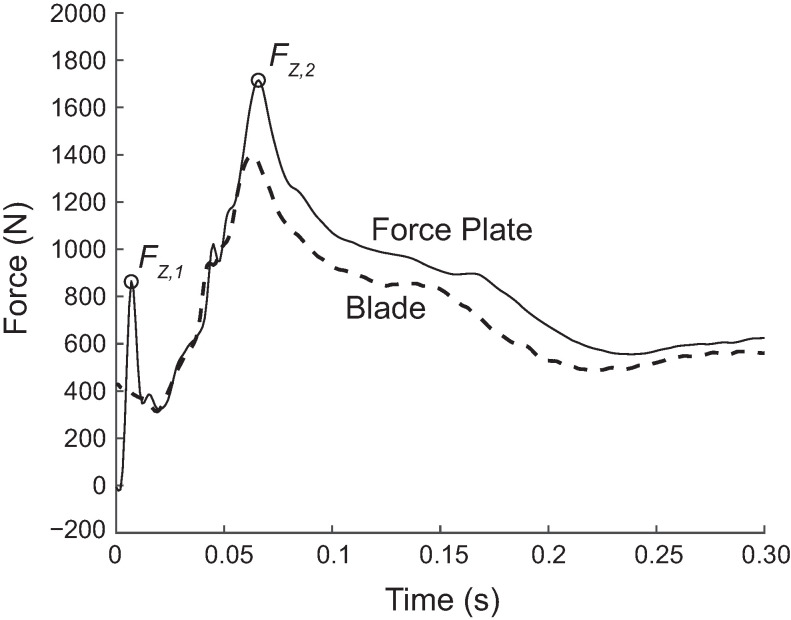
Comparison of forces measured by the force plate and the blade from a representative drop landing. The blade force was calibrated from ten drop/jump landings onto the force plate. The blade force and force plate force typically agreed in overall shape, but disagreed in the presence of the initial impact peak, $F_{Z,1}$, (usually present in the force plate force but not in the blade force), and the height of the second peak $F_{Z,2}$, (usually higher in the force plate force).

None of the peak force measurements were within an acceptable margin of error regardless of the number of landings used in the calibration. The moderate to very strong zero lag correlations suggest that the strain gauge force matched the timing and pattern of the force plate force well. However, it did not identify the initial landing peak ($F_{Z,1}$), where the toe makes contact with the ground (Figure 4). Further, the strain gauge force greatly under- or overestimated (ten-fold) the magnitude of the main peak ($F_{Z,2}$), which occurs when the rest of the blade contacts the ground and the skater’s mass is decelerated. These differences persisted despite testing a variety of calibration matrices, prompting us to consider that the force on the force plate may not be identical to the force on the blade. Elements in series experience the same force in static (and often even quasi-static) loading but not necessarily in dynamic loading. In this case, the strain gauges and the sensing element of the force plate are separated by the blade, the HDPE plastic covering the force plate, and the metal cover of the force plate. These elements have dynamics of their own and may cause the force on the blade stanchions to differ from those on the force plate, especially under high-frequency impacts. In other words, the elements separating the strain gauges and force plate may have absorbed some of the energy of the impact. If the forces on the stanchions and force plate are indeed different during dynamic phases of the landing but similar during quasi-static phases, this calibration method cannot make the force estimated from the strain gauge data match the force plate data over the entire landing. To test this hypothesis, we simultaneously measured the force on a force plate as well as on a load cell rigidly attached to the top of the blade, as described next.

### 2.3. Load Cell Validation

#### 2.3.1. Setup

**Figure 5:**
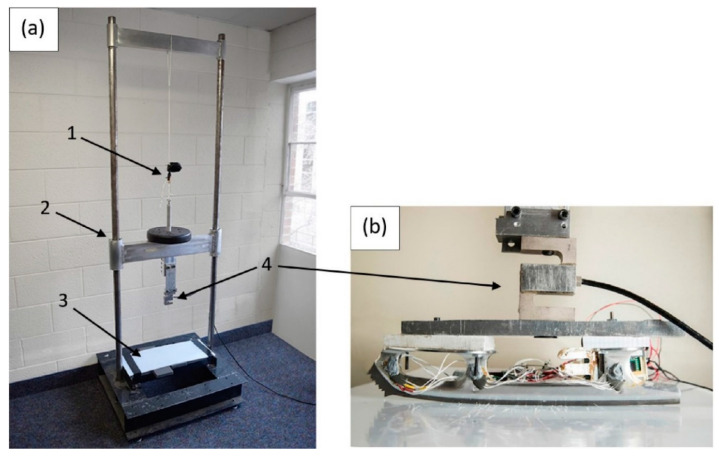
Load cell validation setup. To measure forces under controlled conditions, a drop tower (**a**) was used consisting of a weighted sled mounted on linear bearings (2); the sled was released from different heights using a mechanical archery caliper (1), falling onto a force plate covered by artificial ice (3). Attached to the sled was a load cell (4) and the instrumented blade (**b**).

To measure force with a load cell, we created a controlled setup involving a sled mounted on linear bearings that could be dropped to simulate the impact of landing (Figure 5a). The blade (without the boot) was attached directly to the sled via a manufacturer-calibrated load cell (Omega LCCA-1K, Norwalk, CT, USA) (Figure 5b). The load cell was mounted such that the vertical and horizontal axes of the load cell precisely aligned with the vertical and horizontal axes of the strain gauges. A calibrated force plate (FP4060-05-PT-2000, Bertec Corporation, Columbus, OH, USA) covered by a sheet of UHMW-PE plastic acting as an artificial ice surface was placed at the base of the setup. The hardness of UHMW-PE is of the same order of magnitude as that of ice; the Shore D hardness of UHMW-PE is 60–70, and the Shore D hardness of ice is approximately 50 [14,15]. Load cell and force plate data were collected separately at 1000 Hz via a NI-9327 DAQ system (National Instruments, Austin, TX, USA) and the manufacturer’s software, respectively.

#### 2.3.2. Load Cell vs. Force Plate

**Figure 6:**
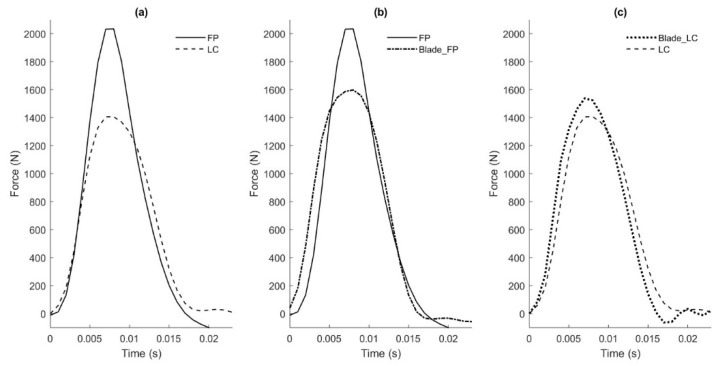
Comparison of force plate (FP) force, load cell (LC) force, blade force from FP calibration (BFP), and blade force from LC calibration (BLC) from one representative controlled drop trial. (**a**) FP and LC forces from the same drop trial, demonstrating the large difference between peak magnitudes; (**b**) FP and BFP forces, showing similarly large differences in peak magnitudes; (**c**) LC and BLC forces, showing better agreement in peak magnitudes.

As mentioned above, none of the force peaks estimated from the strain gauge data matched the force peaks measured by the force plate well, and we hypothesized that this lack of agreement reflected a difference in the forces actually felt by the blade and force plate (see Section 2.2.4 for detailed explanation). If this hypothesis is true, the force plate would be unsuitable for calibrating the blade (at least based on peaks). To test this hypothesis, the force measured by the force plate was compared to the force measured by the load cell. A weight of 90 N was attached to the sled, then the blade was dropped 3 times from a height of 15 cm. Peak forces were determined from the force plate (FP) and load cell (LC). The peak forces from the load cell were 515 ± 107 N (±SD) lower than the force plate (Figure 6a). Since the blade was attached to the load cell but not to the force plate, and since the objects between the blade and load cell (aluminum plates, see Figure 5) were significantly stiffer than the objects between the blade and force plate (plastic and adhesive tape), the force experienced by the load cell was assumed to be a better estimation of the force experienced by the blade. To confirm this assumption, blade force was calculated from the strain gauge data, calibrated with the force plate (Blade_FP) or calibrated with the load cell (Blade_LC). In harmony with the stated assumption, the response estimated from the strain gauges calibrated with the load cell matched the load cell measurement well (Figure 6c), whereas the response estimated from the strain gauges calibrated with the force plate differed from the response of the force plate (Figure 6b). Interestingly, the response estimated from the strain gauges calibrated with the force plate also matched the load cell measurement quite well (compare Figure 6b,c). We interpreted this match as follows: since the force plate data used to calibrate the strain gauges included a substantial quasi-static period (where elements in series experience similar force) and not just dynamic periods (where elements in series do not necessarily experience the same force), the force plate data provided an adequate calibration for the strain gauges. The problem arises when the dynamic periods of the force plate data are used to *validate* the calibration. Since the load cell can be used to both calibrate *and* validate, we continued our investigation with the load cell as reference, testing the effect of (1) loading, (2) blade angle, and (3) excluding horizontal strain gauge data during analysis.

#### 2.3.3. Protocol

Loading: We tested three loading conditions: step, quasi-static, and drop.

The step loading consisted of applying static loads (from approximately 45–222 N) in a step-up and step-down fashion, with each step approximately 10 s in duration. Analysis of initial testing data revealed that calculating the calibration matrix from step loading data resulted in larger peak errors (consistently greater than 200 N) than calculating the calibration matrix from drop loading and therefore was not used in further testing. These large errors are most likely due to the relatively static nature of this loading protocol.

For the quasi-static loading condition, starting with static loads of 201, 401, and 602 N, two investigators applied additional body weight (totaling 1156 N) to the sled, creating a gradual ramp up from static load to maximum load. Ramp up to maximum generally took 3 s, followed by 1–2 s of near-constant weight at maximum loading, and ending with a ramp down of 3 s to the original static load.

The drop loading consisted of dropping the sled with various combinations of heights (between 0.64 and 6.4 cm) and weights (between 45 and 1401 N, in addition to the weight of the 116 N sled) onto the UHMW-PE sheet with foam underneath. Heights, weights, and foam properties were chosen to best replicate the amplitude, shape, and timing of force profiles observed in the off-ice landings performed in the skate. Foam was added to keep the blade from rebounding on the UHMW-PE. Note that although many factors of real landings cannot be replicated in vitro (e.g., how the skater’s body reacts to the impact), matching the force profiles ensures that the strain gauges experienced loading similar to that experienced during real landings.

For all trials, prior to loading, we manually generated a sharp impact for synchronization purposes, followed by 10 s of unloaded data to zero the strain gauges and allow for detrending. A total of 6 to 12 trials were obtained for each of the quasi-static and drop-loading conditions from various height/weight loading combinations.

Blade angle: During on-ice skating landings, IC is made with the posterior portion of the toe pick. As the blade is loaded, the contact area shifts posteriorly, so that at the time of peak force the blade is nearly parallel to the ice. From previous video analysis, it was observed that the center of the contact area was near the middle stanchion, with some variability due to the blade curvature and landing posture. Therefore, we aligned the middle stanchion of the blade in the drop tower to be perpendicular to the impact surface (Figure 5), which placed the contact area just behind the middle stanchion. To test the effects of a more anterior contact area, we tested the blade orientation at a 2.5° anterior tilt, thus covering the approximate range of expected contact points around the occurrence of peak force (from just in front to just behind the middle stanchion).

#### 2.3.4. Data Analysis

The data from the load cell trials were processed using a combination of custom Matlab (Mathworks, Natick, MA, USA) and LabView software. Strain gauge and load cell data were zeroed, detrended, and time synchronized. Calibration matrices were created for each loading condition (quasi-static, drop) from all trials individually using a linear least-squares regression as in the force plate validation described above.

Loading: To test the accuracy of creating calibration matrices from quasi-static loading vs. drop loading, we first predicted the vertical force during each loading condition using an average of the calibration matrices across each loading condition. The two resulting calibration matrices were then applied to each loading condition, resulting in four testing combinations:

1. Quasi-static-loading force predicted using the calibration matrix derived from quasi-static loading,
2. Quasi-static-loading force predicted using the calibration matrix derived from drop loading,
3. Drop-loading force predicted using the calibration matrix derived from quasi-static loading, and
4. Drop-loading force predicted using the calibration matrix derived from drop loading.

To measure out-of-sample performance when the calibration matrix was applied to the same loading type (combinations 1 and 4), we used leave-one-out cross-validation (LOOCV). Nine separate calibration matrices were computed for each loading type.

Blade angle: Calibrations were also tested on data from trials with the blade positioned at the 2.5° anterior tilt to mimic blade angle changes encountered surrounding the occurrence of peak force.

Excluding horizontal strain gauge data: Since most of the force acts in the vertical direction, it may be possible to achieve good force prediction using only the data from the vertical strain gauges. This is particularly important to test at the 2.5° angle. Removing horizontal channels would allow the strain gauges to be configured in a different manner to calculate torque (instead of horizontal force) in addition to vertical force in future designs. Alternatively, the horizontal strain gauges could be removed to allow DAQ channels to be used for other sensors such as accelerometers. To test this possibility, we created calibration matrices using only the 3 vertical channels. This was performed in conjunction with the blade angle tests.

Comparisons between peak forces from the blade and load cell were made using Pearson product moment correlations, root mean square error (RMSE), as well as average and percent differences. An absolute difference of 50 N in peak error was established *a priori* as an acceptable error based on knowledge of typical skater body mass and estimated peak forces.

## 3. Results

**Figure 7:**
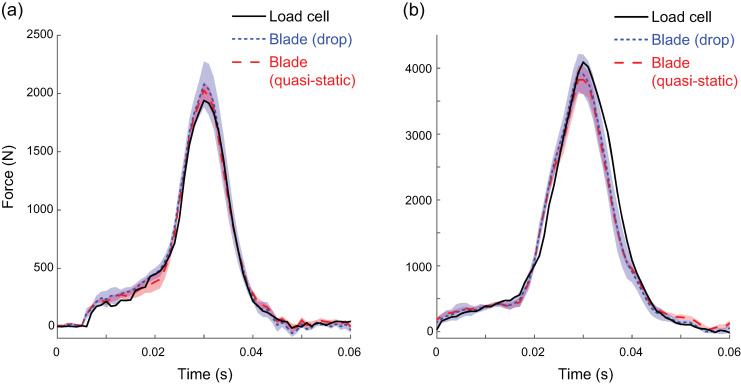
Blade and load cell (LC) forces from two representative drop trials—one with a low weight (**a**) and the other at a high weight (**b**). Blade forces were estimated using either Quasi-static (QS) or Drop (Drop) calibrations. Each of the nine calibration matrices created for each loading condition were applied to the blade data, resulting in nine estimates of force. The lines associated with blade data represent the means of these estimated forces, and the shaded areas represent the standard deviation of the nine estimates of force. In this example, the QS calibration was slightly more accurate on the low force trial and the Drop calibration was slightly more accurate on the higher force trial.

Loading: Calibrating within trial type (i.e., combinations 1 and 4) had peak errors less than 50 N with very strong correlations [16] (Table 1). Calibrating between trial types (combinations 2 and 3) had errors greater than 50 N but still showed very strong correlations. RMS errors were lower for quasi-static trials regardless of calibration method. The main differentiating metric was mean peak force differences, which were lower within trial type. Specifically, mean peak force differences between the load cell and strain gauges in the drop trials were 6 N underestimated for the drop-loading calibration and 131 N underestimated for the quasi-static loading calibration. All peak differences were within the *a priori* established 10% difference. Exemplar force profiles from two drop trials are provided in Figure 7.

Blade angle and excluding horizontal strain gauge data: When calibrating with three channels (only vertical) versus six channels (three vertical + three horizontal) with the blade flat, there were very strong correlations between the load cell and blade with peak errors <50 N (<2% difference). With the blade tilted, correlations remained very strong but absolute peak differences increased and were >50 N. Despite absolute peak differences near 175 N with the tilted blade, percent differences remained less than 10%. The average percent difference was <1% with the blade flat and <7% with blade tilted using all six channels (Table 2). Therefore, we chose to include all three channels of data in further analyses.

### On-Ice and Off-Ice Application

**Figure 8:**
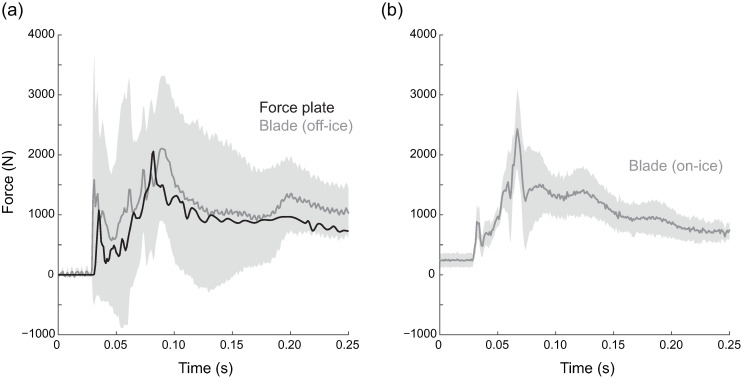
Drop-loading calibration applied to two example jumps. (**a**) Off-ice jump from a box onto a force plate. The blade force roughly matches the force plate force in timing and magnitude. (**b**) On-ice double flip jump, for which there is no force plate equivalent. The peak values are similar to the off-ice jump, but the initial toe contact peak is much smaller. The shaded areas represent the standard deviation of the force estimates calculated from the nine drop trial calibration matrices.

To preview potential applications, we collected preliminary data from a senior-level competitive figure skater performing an off-ice landing and an on-ice jump (approved by the Brigham Young University Human Subjects Review Board, study #15304). The off-ice landing was performed from a box onto a force plate (FP4060D-05, Bertec, Columbus, OH, USA) following the same procedures (hopping backwards from a box onto the force plate) used in the force plate validation section. The force plate was used only to compare general shape and timing of force events, as we previously determined that the force plate and blade experience different dynamic loads (see above). For the on-ice jump, the skater performed a double flip: the skater took off backward, starting on her non-dominant foot, while the toe pick of the dominant foot was used to vault the skater into the air. Two rotations were performed in the air followed by a single leg landing on the dominant foot. The drop calibration procedure detailed above was applied to the strain gauge data from both the on-ice and off-ice jumps. The blade data from the box jump matched the magnitude and shape of the force plate data quite closely (Figure 8a). On-ice data (Figure 8b) was similar in magnitude and shape to the off-ice jump, but the initial toe contact peak was much smaller.

## 4. Discussion

The controlled drop method of calibrating the strain gauges resulted in errors for predicted impact peaks that were lower than 10% when applied to other controlled drops. This method is also easier to duplicate than the QS condition and, therefore, is the method that will be used in future iterations of this project. Using the drop calibration method, preliminary measurement of on-ice forces for a double flip jump were approximately 4 times body weight, which is on the lower end of several previous indirect estimates of on-ice landing forces [6,10,11,17].

Instrumented blade calibration for on-ice force measurements is challenging due to the difficulty in establishing a gold standard reference. Initially, it seemed logical to use off-ice landings for calibration, assuming that these landings would have characteristics similar to on-ice jump landings. However, it was difficult to achieve sufficiently high peak forces without substantially raising the box to impractical heights. Varying the combination of drop height, weight, and foam thickness during controlled drops of the blade on a drop tower resulted in a larger and more appropriate range of impact characteristics. We also determined that the force plate would not serve as an acceptable gold standard, even during the controlled drops. Comparisons between force plate and load cell data from these trials showed discrepancies which we attributed to the interaction of the landing surface and blade during impact.

Matching specific aspects of the force waveform during impact was also difficult. Part of this was due to the presence of two impact peaks, which is common when landing kinematics include making initial ground contact with the ball of the foot [18]. During the skaters’ landings onto the force plate, the initial impact peak was particularly troublesome (Figure 4, $F_{Z,1}$). In most cases, this initial peak was not present in any of the individual strain gauge channels. It is possible that this force is present at the force plate, but not at the blade due to the different dynamic loading properties of the in-series elements. Subsequently, including the initial peak in the calibration process resulted in reduced accuracy on the second impact peak ($F_{Z,2}$). This hurdle prompted us to investigate further using drop tests. During controlled drop testing comparisons, both the force plate and load cell were able to capture the general shape of the force curves. However, the load cell appeared to better align with the strain gauge responses during the second impact peaks. We therefore decided to focus on obtaining the second peak using a controlled drop (and load cell) for calibration. Previous research has shown that this peak is typically higher than the first when the ankle goes through a limited range of motion, as it does in figure skating boots [18]; therefore, this second peak is likely of greater importance from an injury standpoint [6]. In addition to peak force, loading rate (slope of the second peak) is also a likely contributor to both acute and chronic injuries [5]. A critical future on-ice application will be to study the relationship between loading rate and injury during figure skating jump landings. While we focused on obtaining peak forces in this study, the accurate measurement of peak forces along with their timing will allow us to quantify loading rate.

Although there are a number of challenges in applying a controlled calibration to uncontrolled conditions, a number of results showed promise in accurately estimating on-ice force metrics.

Calibration results from both force plate and load cell tests showed that accuracy in estimating curve shape and the second impact peak (Figure 4, $F_{Z,1}$) was quite good when applied to similar loading conditions. However, on-ice jumps cannot be perfectly duplicated in a controlled environment. The amount of torque generated as a skater lands from a rotational jump is currently unknown and therefore difficult to simulate. The off-ice results indicated that the largest peak force during a skating landing occurs when the blade is parallel to the ice. The flat blade position and drop test calibration procedure appeared to capture this key force metric. We also noted that during test landings made by a skater wearing the instrumented blade, the strain gauges on the middle blade stanchion generally had the highest strains, suggesting that most of the skaters’ effective mass was positioned over the middle to front part of the skate. This was slightly surprising since it has long been assumed that the peak forces in most drop landings occur when the heel makes contact with the ground [19]. Despite the differences in the dynamics of an on-ice jump compared to an off-ice simulated landing, the data from the on-ice force measurement were encouraging. For instance, the peak force from the double flip jump was similar in magnitude and timing to the off-ice drop jumps. It is unclear whether an initial impact peak occurred during this on-ice jump or whether the IceSense did not capture it. Overall, the presented curve represents the first measured on-ice jump landing force.

Designing and implementing this on-ice force measurement device included significant challenges due to the on-ice environment and impact dynamics of figure skating jumps. A number of design improvements were made to make the previously described system [13] more accurate and robust (see Supplementary Material for details). Nevertheless, the IceSense device still includes a number of limitations that would need to be overcome if this type of device were to be more widely used by other scientists or coaches. First, despite much effort to make the system more robust, it remains vulnerable to impact; to improve the robustness, the electronics should be more fully integrated in a single printed circuit board to solidify electrical connections and include damping features to reduce high frequency vibrations. Second, in its current state, the system only measures forces in the sagittal plane of the blade but does not measure force in the medial–lateral direction or any torques. Third, since it is not currently possible to measure on-ice forces with any other device, the performance of the IceSense on ice has not been validated directly. Finally, the blade was calibrated in a lab at room temperature but will be used in a rink at colder temperatures. We tested the effect of this change in temperature on the calibration (see Supplementary Material). Although the change in temperature did not appear to affect the shape of the response or the accuracy of the calibration, it did cause an offset in the estimated force. Additional procedures were required to avoid sensor saturation (adjusting the electronics gain once the system had adjusted to the temperature on ice) and remove the offset (subtract the measured force during the flight phase).

The IceSense proof-of-concept and calibration procedure were encouraging in estimating key measures related to figure skating injuries and performance. Although not without its own limitations, the instrumented blade overcomes many of the prohibitive hurdles associated with previous devices. It should be noted that the IceSense proof of concept presented in this paper is the accumulated result of several years’ worth of testing, and newer electronic components are likely available that may allow for additional miniaturization of the IceSense framework. It is also possible that pressure insoles or load cell technology will improve in the coming years and allow for compatibility with skating boots, and even integration with IceSense. Currently, we plan on using IceSense for research purposes as detailed in this paper (with updated components) to analyze on-ice skating jumps. Accurate measurement of on-ice forces has the potential to increase understanding of the relationships between impact force and injury development. While the focus of this study was landing forces, injuries may also be related to take off [20,21], and the IceSense could be used to measure forces during take off as well. This information could also influence future boot and blade designs as well as jumping and landing technique adjustments. Additionally, further development of this system may allow instrumented blades to be used to monitor training volume (i.e., jump count), performance parameters (e.g., jump height), and fatigue.

## 5. Conclusions

Accurate on-ice force estimation has the potential to increase the understanding of overuse injuries in figure skating and influence training strategies as well as injury prevention and rehabilitation protocols. This study suggests that using a controlled drop method to calibrate the strain sensors against a load cell mounted above the skate blade provides the most representative force data during off-ice jump landings. Data presented from a single on-ice jump landing indicate that this system could provide important information when used in future figure skating research.

## Figures and Tables

**Table 1:** Comparison of measured load cell force and calculated blade force using calibrations from two loading conditions (quasi-static versus drop) and one calibration calculation variety (8-in-1-out drop calibration) on nine drop trials (mean ± stdev).

	Quasi-Static Trials		Drop Trials	
	QS Calibration	Drop Calibration	QS Calibration	Drop Calibration
r-value	1.0	1.0	0.99	0.99
RMSE (N)	13.7 ± 5.4	39.7 ± 9.3	63.6 ± 45.0	59.1 ± 33.3
Peak Diff (N)	14.6 ± 13.5	78.8 ± 14.5	−131.3 ± 275.5	−6.2 ± 277.6
Peak Diff (%)	0.8 ± 0.9	5.5 ± 0.7	−2.9 ± 7.4	1.4 ± 8.2

**Table 2:** Effects of blade tilt on blade force accuracy. The blade calibration was created from flat trials using signals from the three channels representing vertical strain or from all six channels representing vertical and horizontal strain. These calibrations were then applied to both flat and tilted trials (2.5° anterior tilt).

	Flat		Tilt	
	Vertical Strain (z)	Vertical (z) and Horizontal (x) Strain	Vertical Strain (z)	Vertical (z) and Horizontal (x) Strain
r-value	0.99	0.99	0.99	0.99
RMSE (N)	58.0 N	58.3 N	45.4 N	45.6 N
Avg Peak Diff (N)	14.0 N	−18.4 N	172.8 N	173.8 N
Avg Peak Diff (%)	1.5%	0.9%	9.0%	6.6%

## Supplementary Materials

The supplementary materials are available online at https://www.mdpi.com/1424-8220/20/24/7082/s1. These supplementary materials provide detailed information about the improvements to the wiring, Wheatstone bridge boards, signal conditioning board, and protection of the electronics. We also provide the results of testing the temperature sensitivity of the device.
