# Supplementary material for: IceSense Proof of Concept: Calibrating an Instrumented Figure Skating Blade to Measure On-Ice Forces
Source: Sensors (Basel). 2020 Dec 10;20(24):7082. doi: 10.3390/s20247082 (PMC7763340; doi:10.3390/s20247082)
Supplement: Supplementary file 1 [file sensors-20-07082-s001.pdf]

sensors

MDPI

Supplementary Material for: IceSense Proof of Concept: Calibrating an Instrumented Figure Skating Blade to Measure On-Ice Forces

Sarah Ridge [1,*], Dustin Bruening [1], Steven Charles [2], Cody Stahl [3], Daniel Smith [2], Riley Reynolds [2], Brandon Adamo [2], Blake Harper [2], Chris Adair [2], Preston Manwaring [4] and Deborah King [3]

[1] Department of Exercise Sciences, Brigham Young University, Provo, UT 84602 USA; dabruening@byu.edu
[2] Department of Mechanical Engineering, Brigham Young University, Provo, UT 84602 USA; skcharles@byu.edu (S.C.); dannysmith@gatech.edu (D.S.); rileyreynolds1218@gmail.com (R.R.); jbadamo14@gmail.com (B.A.); blake.harper@byu.edu (B.H.); chriswadair@byu.edu (C.A.)
[3] Department of Exercise Science and Athletic Training, Ithaca College, Ithaca, NY 14850 USA; cstahl@uccs.edu (C.S.); dking@ithaca.edu (D.K.)
[4] Blue Elm Consulting, Farmington, UT 84025 USA; preston.k.manwaring@dartmouth.edu
* Correspondence: sarah_ridge@byu.edu

1. Design Improvements

Since publishing the initial design of the IceSense on-ice force sensing system [13], important design improvements have been made to increase the signal quality and the robustness of the measurements. These improvements included re-routing the wiring from the power supply, modifying the design of the Wheatstone bridge board and circuitry, raising the gain of the signal conditioning board, and improving the protection of the electronics. Each improvement is described in detail below.

Wiring (Figure 1)*:* In the initial wiring scheme, only the data logger was directly connected to the battery. Power was then fed from the data logger to the rest of the boards. While this wiring scheme was simple and in principle ensured that there was a common ground and power provided to each board, the additional power running through the data logger damaged its components; it burned the tracing that carried power through the data logger and distributed it to other boards. To remedy this problem, each board was connected directly to the battery. Although the new system was more cumbersome, it protected the electronics since no board was being used to distribute power to other boards. In addition, all boards were connected to ground at a single point (star grounding) to isolate the analog signal from noise in the digital components, reducing an artifactual periodic spike in the output signal.

Wheatstone Bridge Boards (Figure 1c)*:* In the initial data acquisition system, the bridge load regulation from the voltage regulator was noisier than expected. This impacted the accuracy of the data acquisition circuitry at the micro-volt level. The simplest solution was to substitute a high-precision voltage reference source capable of driving the bridge circuit. A REF195 low-dropout voltage regulator (Analog Devices, Norwood, MA) accurate to 4 ppm/mA and 5 ppm/C was used. Further, a vibration-stable trimming resistor was incorporated into the circuit to zero the output of the bridge, thus maximizing the signal available to the data acquisition circuitry. Bridge trimming was performed after temperature stability was achieved prior to each collection session.

Signal Conditioning Board (under data logger shown in Figure 1d)*:* To increase the sensitivity of the system, the gain of the signal conditioning board was raised. This gain can be set separately for each channel and depends on the gain resistor of each channel: $G = (49.9\ k\Omega/R_g) + 1$, where G is the gain and R_g is the resistance of the gain resistor. The initial system had gain resistors of 604 Ω, resulting in a gain of 83.6. By testing multiple gain resistors while skaters performed jumps on ice, it was determined that a gain resistor of 50 Ω and the resulting gain of 999 maximized the sensitivity of the system while still avoiding saturation.

Protecting the Electronics (Figures 1f–g)*:* Using the system off ice and on ice, vibrations (as occur during a landing, for example) often caused channels to shift and re-zero. These vibrations affected the electronic boards and wiring. To reduce the movement of the wires and electronics relative to the boot, the Wheatstone bridge boards were secured to their respective stanchions using tape. Second, the bracket was redesigned to orient the signal conditioning board downward, allowing the wires to be bundled in wire wrap, connected to the signal conditioning board, switch and LED, and taped to the boot and blade to avoid movement against the blade. Care was taken not to secure the wires too tightly, as this was found to cause damage at the connections between boards.

The electronics are sensitive to water and ice. A boot cover was designed from 3 mm thick neoprene, which is both waterproof and can be stretched to fit tightly around the boot. The bottom portion of the cover was glued to the blade below the stanchions using Hi-Strength 90 Spray Adhesive (3M, St. Paul, MN, USA), which bonded the neoprene to the portion of the aluminum blade holder immediately above the steel blade, protecting all the electronics. The top and back portions of the neoprene panels were joined using Velcro to enable easy access to all electronics.

2. Temperature Sensitivity

The stanchions and strain gauges (Figure 1b) also respond to changes in temperature. Two different tests were performed to analyze the system's sensitivity to these changes. In the first test, a thermocouple was attached to the middle stanchion (next to one of the strain gauges) and measurements were recorded under three different conditions: room temperature (approximately 23 °C), cold (next to the ice, approximately 7–10 °C), and in contact with the ice (approximately 3–6 °C). Under each of these conditions, voltage was recorded as a static, vertical force of 100 lb was applied to the blade after it had reached thermal equilibrium. The results showed that the different conditions caused an offset in voltage but did not otherwise affect the response.

In the second test, the effect of temperature on calibration accuracy was determined during the force plate validation (see Force Plate Validation above). One calibration matrix was generated from 10 landings with the blade at ice temperature and another calibration matrix from 10 landings with the blade at room temperature. Both calibrations were then applied to 118 landings performed with the blade at ice temperature. To get the blade to ice temperature, the boot-blade system was placed on a 20 cm by 25 cm sheet of ice in a Styrofoam cooler for 25 minutes prior to testing. Between landings, the skater stood on the ice sheet for 20 to 30 seconds, which proved sufficient to maintain the blade at ice temperature. In analyzing the results, we found that temperature did not affect calibration accuracy (results not shown for brevity).

The results of both tests together led to the conclusion that blade temperature changed the baseline values of each channel, but do not affect the strain gage channel responses. To avoid sensor saturation, this change in baseline (caused by the change from room temperature to near-ice temperature) was counteracted by trimming the Wheatstone bridges once the system had equilibrated to the near-ice temperature.
